# Reduced-Dose Rivaroxaban Is Associated with Lower All-Cause Mortality in Older Patients with Nonvalvular Atrial Fibrillation

**DOI:** 10.3390/jcm12206686

**Published:** 2023-10-23

**Authors:** Wei-Ru Chiou, Min-I Su, Ying-Hsiang Lee, Po-Lin Lin, Cheng-Wei Liu

**Affiliations:** 1 Division of Cardiology, Taitung MacKay Memorial Hospital, Taitung 95054, Taiwan; clementchiou@gmail.com (W.-R.C.); wwfsone@gmail.com (M.-I.S.); 2 Department of Medicine, MacKay Medical College, New Taipei 25245, Taiwan; 3 Cardiovascular Center, MacKay Memorial Hospital, Taipei 10449, Taiwan; 4 Department of Artificial Intelligence and Medical Application, MacKay Junior College of Medicine, Nursing, and Management, Taipei 11260, Taiwan; 5 Department of Nursing, MacKay Junior College of Medicine, Nursing, and Management, Taipei 11260, Taiwan; 6 Division of Cardiology, Hsinchu MacKay Memorial Hospital, Hsinchu 30046, Taiwan; 7 Division of Cardiology, Department of Medicine, Kuang Tien General Hospital, Taichung 43302, Taiwan; 8 Department of Nutrition, Hungkuang University, Taichung 433304, Taiwan

**Keywords:** rivaroxaban, atrial fibrillation, mortality, elderly

## Abstract

Background: Reduced-dose rivaroxaban (10 mg) was used in the J-ROCKET AF trial, demonstrating safety in the Asian population. It remains unclear whether treatment with reduced-dose versus full-dose rivaroxaban (20 mg/15 mg) is associated with all-cause mortality in older patients with nonvalvular atrial fibrillation. Proposed: To evaluate the effects of reduced-dose rivaroxaban on all-cause mortality in patients over 85. Methods: We retrospectively enrolled medical records representing the period from October 2012 to November 2016. The 2 × 2 factorial design incorporated age (≥85 vs. <85) and rivaroxaban use (reduced vs. full dose). The primary study outcomes were all-cause and cardiac-related mortality. Results: The study enrolled 2386 patients with a mean age of 76.6 ± 10.4 years; 51.8% were male. In the ≥85 group (*n* = 593), the reduced-dose subgroup had lower all-cause (5.3% vs. 10.6%, *p* = 0.02) and cardiac-related mortality (1.9% vs. 5.1%, *p* = 0.04), whereas the younger patients receiving reduced-dose rivaroxaban had higher all-cause mortality (3.7% vs. 1.8%, *p* = 0.01) but no difference in cardiac-related mortality (1.2% vs. 0.7%, *p* = 0.33). The rate of hospitalization for heart failure was significantly lower in the elderly group with reduced-dose rivaroxaban (7.2% vs. 15.7%, *p* < 0.01) but not in the younger group. After adjusting for confounders in the older group, treatment with reduced-dose rivaroxaban was associated with lower risk of all-cause mortality (adjusted HR (aHR): 0.40, 95% CI: 0.21–0.74, *p* < 0.01) and hospitalization for heart failure (aHR: 0.54, 95% CI: 0.29–0.99, *p* = 0.05). No associations were found between rivaroxaban dose and cardiac-related mortality in either group, nor between younger age and any outcome. Conclusions: Reduced-dose rivaroxaban was associated with lower risks of all-cause mortality and hospitalization for heart failure in older patients with nonvalvular atrial fibrillation. Future studies can investigate the effect of reduced-dose rivaroxaban on prognoses in elderly individuals ≥85 years in the west.

## 1. Introduction

Direct oral anticoagulants (DOACs) are associated with decreased risks of ischemic stroke or systematic embolization in patients with nonvalvular atrial fibrillation [1]. Although the risks of ischemic stroke are attenuated in these populations by DOAC usage, bleeding complications may still occur in patients with atrial fibrillation, especially in patients with comorbidities such as old age, chronic kidney disease, and uncontrolled hypertension [2,3,4]. A systematic review and meta-analysis showed that DOAC usage was associated with a trend toward a decreased risk of all-cause mortality in four major randomized controlled clinical trials [1,5,6,7,8]. The risk of ischemic stroke in patients with atrial fibrillation is stratified by baseline characteristics and increases as patients become older. A gap is seen between prognoses and DOAC use in elderly patients with atrial fibrillation. Although the full dose of DOAC provides comparable efficacy for stroke prevention in nonvalvular atrial fibrillation patients with a reduced risk of bleeding complications [6], the safety of rivaroxaban remains uncertain in patients older than 85 years.

We previously published a study to discuss the association between rivaroxaban and clinical outcomes [9]. Therefore, we conducted the present study retrospectively to investigate the efficacy and safety of full- versus reduced-dose rivaroxaban in atrial fibrillation patients aged more or less than 85 years.

## 2. Materials and Methods

**Figure 1:**
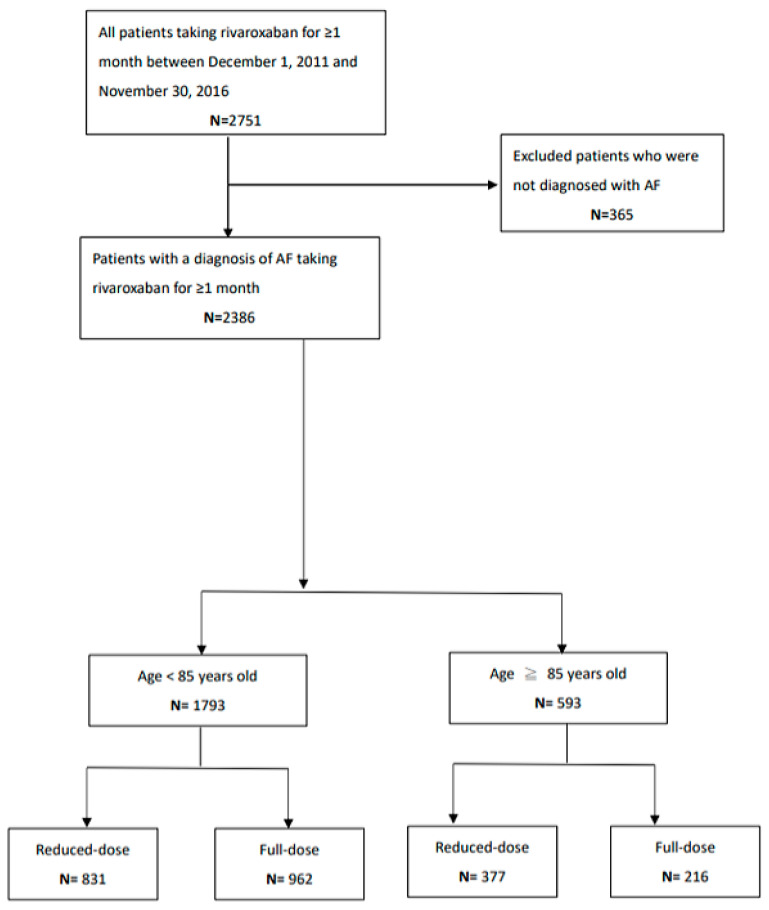
Flow diagram of patient selection.

We conducted the present study and retrospectively collected patient data from medical records, including baseline characteristics and laboratory data as well as indication and dose of rivaroxaban between 1 December 2011 and 30 November 2016. The patients were from the four hospitals of the MacKay system, including Taitung, Tai5pei, Tamsui, and Hsinchu Memorial Hospital. We followed all of our study outcomes cross-sectionally for one year, with the last follow-up date being the end of December 2017. Our study was approved by the MacKay institutional review board (number 16MMHIS009). Given that the present study was a retrospective cohort study and of low risk to the study patients, the board waived informed consent in our study. Regarding ethnicity differences, the lower dosage of 15 mg rivaroxaban was used as the full dosage in some areas of Asia such as Japan [10]. We classified the study patients by use of 20/15 mg and 10 mg of rivaroxaban into the full- and reduced-dose groups on the first day of rivaroxaban prescription. The study flow chart is shown in Figure 1. Because the pathophysiologies and the dosages of rivaroxaban were somewhat different between patients with nonvalvular atrial fibrillation and venous thromboembolism, we only enrolled atrial fibrillation patients with rivaroxaban use. We calculated the atrial fibrillation-related stroke risk according to the well-acknowledged model CHA2DS2Vsc score [11]. We used the other model, namely, the HASBLED score [12], to stratify the risk of bleeding complications induced by DOACs.

The primary efficacy outcomes were all-cause mortality and cardiac-related mortality, and the incidence of clinically relevant bleeding complications, including intracranial hemorrhage, gastrointestinal bleeding, a drop in the hemoglobin value of 2 g/L or more, and bleeding complications requiring surgical intervention, was the primary safety outcome. The secondary efficacy outcomes included hospitalization for heart failure, nonfatal myocardial infarction, and ischemic stroke; gastrointestinal bleeding was a secondary study outcome.

In statistical analyses, we expressed continuous variables as numbers and standard deviations (SD) and binary variables as numbers and percentages; we used independent *t*-tests to evaluate differences in continuous variables, and binary variables were evaluated by chi-squared tests. We first classified the patients into two groups according to the age threshold of 85 years; the baseline characteristics and laboratory data are presented in Table 1. We used univariate logistic regression analyses to evaluate the association between the reduced- vs. full-dose rivaroxaban and the study outcomes; we considered the variables as confounders if they were significantly associated with the study outcomes in univariate logistic regression analyses. If a significant association between the rivaroxaban dose and a study outcome existed in univariate logistic regression analysis, we adjusted for confounders to investigate the actual association between the rivaroxaban dose and the study outcomes in multivariate logistic regression analyses. All *p* values are two-tailed, and *p* values of 0.05 or lower are considered significant. We performed all statistical analyses with the Statistical Package for the Social Sciences (SPSS software, version 20.0).

## 3. Results

The study cohort consisted of 2386 patients, 51.8% of whom were male, with a mean age of 76.6 years (SD 10.5), CHA2DS2Vsc score of 4.4 (SD 1.7), and HASBLED score of 1.6 (SD 1.0). In the overall study population, the older patients aged ≥85 years (*n* = 593) versus the patients aged < 85 years (*n* = 1.793) were predominantly female (59% vs. 46%, *p* < 0.01) and had a higher prevalence of history of hospitalized heart failure (10.3% vs. 6.6%, *p* < 0.01) and previous ischemic or transient ischemic stroke (31.2% vs. 25.4%, *p* < 0.01), as well as a greater rate of uncontrolled hypertension with blood pressures greater than 160 mm Hg (9.3% vs. 6.3%, *p* = 0.02); they had lower values of diastolic blood pressure (72.7 (11.9) mm Hg vs. 75.9 (12.1) mm Hg, *p* < 0.01) and estimated glomerular filtration rate (61.4 (25.7) mL/min/1.73 m^2^ vs. 68.9 (25.8) mL/min/1.73 m^2^, *p* < 0.01), as shown in Table S1. The overall study population was classified into reduced- vs. full-dose groups; the two groups had significantly different occurrence rates related to types of atrial fibrillation, medical history of permanent pacemaker implantation, and medications for chronic cardiovascular disease, except that the prescription rates of nonsteroid anti-inflammatory drugs were similar in the two groups, as shown in Table S2. We classified the patients by the age of 85 years and compared the incidence of events with respect to treatment with reduced- vs. full-dose rivaroxaban. In patients aged ≥85 years, the reduced-dose group had lower rates of a history of hospitalization for heart failure (reduced dose 43.0% vs. full dose 51.9%, *p* = 0.04), major bleeding complications (4.2% vs. 18.1%, *p* < 0.001), an international normalized ratio > 3 (1.3% vs. 12.5%, *p* < 0.001), and diabetes mellitus (26.3% vs. 32.9%, *p* = 0.09), whereas the rates of uncontrolled hypertension (11.1% vs. 6.0%, *p* = 0.04) and chronic kidney disease (30.2% vs. 18.1%, *p* = 0.001) were significantly higher in the reduced-dose group. In the younger group (age < 85 years), the reduced-dose group had significantly lower rates of a history of major bleeding complications (reduced dose 3.7% vs. full dose 11.3%, *p* < 0.001), systematic embolization (24.3% vs. 32.4%, *p* < 0.001), ischemic stroke (20.2% vs. 29.8%, *p* < 0.001), an international normalized ratio > 3 (1.3% vs. 9.2%, *p* < 0.001), and the use of an antiplatelet agent or nonsteroidal anti-inflammatory drugs (9.9% vs. 17.9%, *p* < 0.001). The prevalence of chronic kidney disease was significantly higher in the reduced-dose group (21.1% vs. 10.4%, *p* < 0.001). The prescription rates of antiarrhythmic agents and other medications for cardiovascular diseases were quite different between the two age groups, but the use of aspirin was not significantly different between the older and younger patients of either the reduced- or full-dose group. Table 1 shows the baseline characteristics between the two groups.

**Figure 2:**
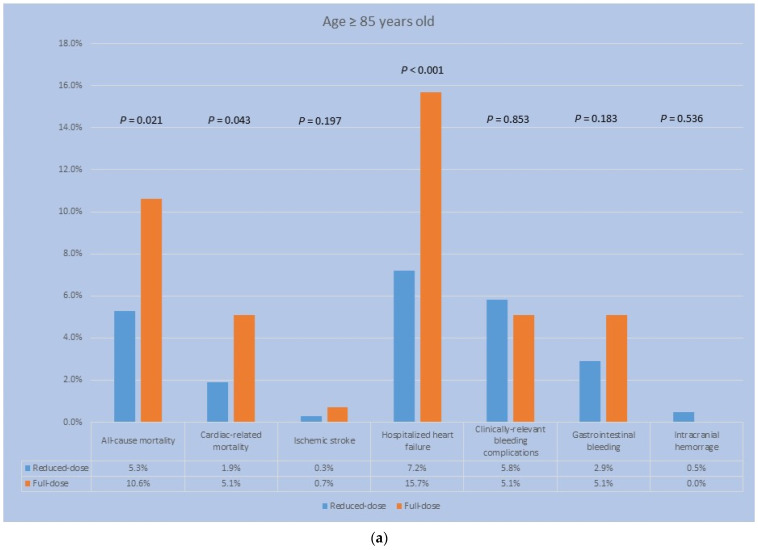

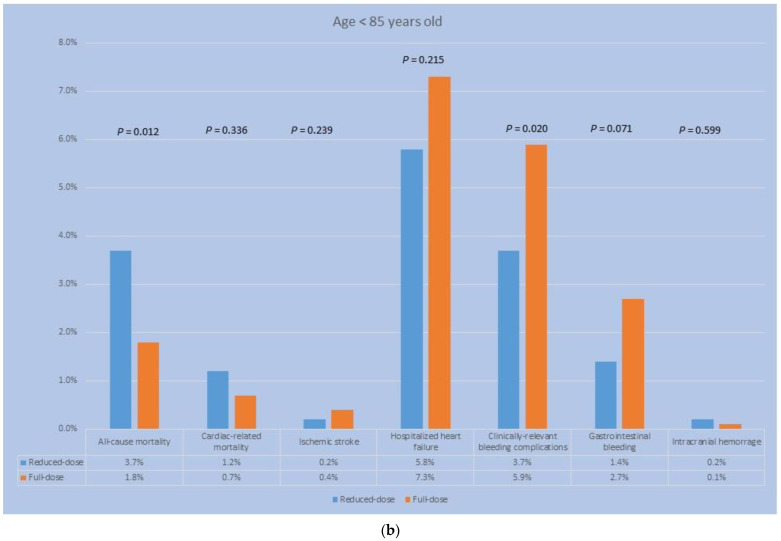
(**a**) The patients aged 85 years or more receiving reduced-dose rivaroxaban had significantly lower incidences of all-cause and cardiac-related mortality, as well as hospitalized heart failure, than patients receiving the standard dose. The rates of bleeding complications were not significantly different between the two groups. (**b**) The patients aged less than 85 years receiving reduced-dose rivaroxaban had a significantly greater all-cause mortality compared with patients receiving the standard dose. No significant differences were found regarding cardiac-related mortality and hospitalized heart failure. Bleeding complication rates were significantly lower in the patients receiving lower doses of rivaroxaban.

**Figure 3:**
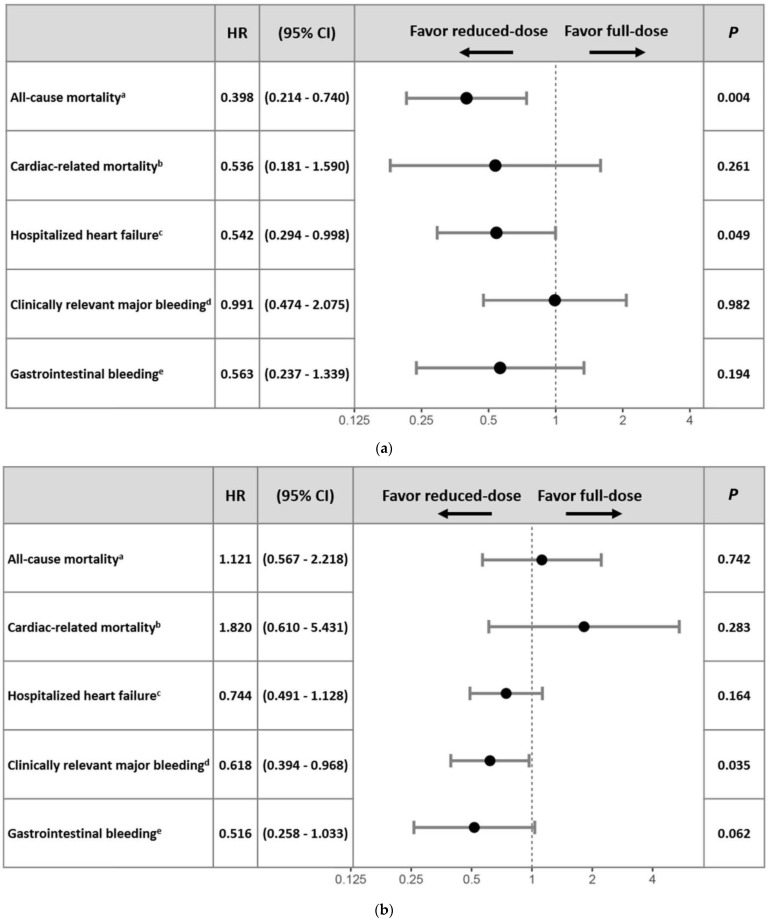
(**a**) Multivariate logistic regression analyses of age ≧ 85. ^a^ Adjusted variable: rivaroxaban dose, age, sex, history of hypertension, and usage of ACEI or ARB. ^b^ Adjusted variable: rivaroxaban dose, age, sex, history of heart failure admission, creatinine, left atrium diameter, and AF classification. ^c^ Adjusted variable: rivaroxaban dose, age, sex, vascular disease, AF classification, history of heart failure admission, history of major bleeding, labile INR, vascular disease, ticlopidine usage, and cilostazol usage. ^d^ Adjusted variable: rivaroxaban dose, age, sex, left atrium diameter, and hemorrhage stroke. ^e^ Adjusted variable: rivaroxaban dose, age, sex, and creatinine. (**b**) Multivariate logistic regression analyses of age < 85. ^a^ Adjusted for rivaroxaban, age, gender, systolic blood pressure, diastolic blood pressure, creatinine, eGFR, AF classification, history of heart failure admission, any stroke, usage of ACEI or ARB, usage of statin, and CKD more than stage IIIb. ^b^ Adjusted variable: rivaroxaban dose, age, sex, usage of clopidogrel, usage of ticagrelor, eGFR, history of heart failure admission, CKD more than stage IIIb, and labile INR. ^c^ Adjusted variable: history of diabetes mellitus, usage of clopidogrel, usage of ticagrelor, GPT, systolic blood pressure, creatinine, eGFR, vascular disease, usage of aspirin, Af classification, history of heart failure admission, CKD more than stage IIIb, and usage of antiarrhythmia drug. ^d^ Adjusted variable: rivaroxaban dose, age, sex, clopidogrel usage, ticagrelor usage, and ticlopidine usage. ^e^ Adjusted variable: rivaroxaban dose, age, sex, and usage of ticlopidine.

With respect to the study outcomes in the older group, the patients in the reduced-dose rivaroxaban group had a lower incidence of all-cause mortality (5.3% vs. 10.6%, *p* = 0.02), cardiac-related mortality (1.9% vs. 5.1%, *p* = 0.04), and hospitalized heart failure (7.2% vs. 15.7%, *p* < 0.01), compared with patients in the full-dose group, whereas the incidence of all-cause mortality was not significantly different between the reduced- and full-dose rivaroxaban groups (5.7% vs. 4.4%, *p* = 0.231) and neither were cardiac-related mortality (1.2% vs. 0.7%, *p* = 0.33) and hospitalized heart failure (5.8% vs. 7.3%, *p* = 0.21) in patients aged <85 years old. The incidence of ischemic stroke was not significantly different in patients ≥ 85 years old (0.3% vs. 0.7%, *p* = 0.197) and patients aged <85 years old (0.2% vs. 0.4%, *p* = 0.239). Regarding bleeding complications, the rates of incidence were not significantly different between the older and younger patients in the two groups, except that the incidence of any bleeding was significantly lower in the patients with the reduced- vs. full-dose rivaroxaban in the younger group (shown in Figure 2 and Figure 3).

Considering the primary efficacy outcomes, treatment with reduced- vs. full-dose rivaroxaban was associated with decreased risks of all-cause mortality (crude hazard ratio (cHR): 0.49, 95% confidence interval (CI) 0.27–0.89, *p* = 0.02) and cardiac-related mortality (cHR: 0.36, 95% CI: 0.14–0.93, *p* = 0.03) in patients aged more than 85 years (shown in Figure 3a). In contrast, reduced- vs. full-dose rivaroxaban treatment was associated with increased risks of all-cause mortality (cHR: 2.14, 95% CI: 1.18–3.87, *p* = 0.01) but not cardiac-related mortality (cHR: 1.66, 95% CI: 0.63–4.4, *p* = 0.30) in the younger group (shown in Figure 3b). Considering the secondary efficacy outcomes, reduced- vs. full-dose rivaroxaban treatment was associated with a lower risk of hospitalized heart failure (cHR: 0.44, 95% CI: 0.26–0.72, *p* < 0.01) in the older group, but a significant association was not found among younger patients (cHR: 0.80, 95% CI: 0.55–1.15, *p* = 0.23). Patients in the reduced-dose and full-dose groups did not have significantly different risks for nonfatal myocardial infarction or stroke, whether older or younger. With respect to the primary and secondary safety outcomes, the reduced- vs. full-dose rivaroxaban treatment had no significantly different association in the older and younger groups, except that a significant association was found between reduced-dose treatment and clinically relevant bleeding complications in the younger group (cHR: 0.63, 95% CI: 0.40–0.97, *p* = 0.04).

In the univariate logistic regression analyses, we examined the associations between several variables and different study outcomes among the patients aged more than 85 years. The variables associated with the all-cause mortality were rivaroxaban dose (standard vs. low dose) (cHR: 2.04, 95% CI: 1.12–3.71, *p* = 0.02), age (cHR: 1.09, 95% CI: 1.02–1.17, *p* = 0.11), sex (female vs. male) (cHR: 1.07, 95% CI:0.58–1.97, *p* = 0.83), history of hypertension (cHR: 0.51 95% CI: 0.27–0.94, *p* = 0.03), and usage of ACEI or ARB (cHR: 0.49, 95% CI: 0.27–0.88, *p* = 0.02). The association between the cardiac-related mortality and the variables included rivaroxaban dose (cHR: 2.78, 95% CI: 1.08–7.18, *p* = 0.03), creatinine (cHR: 0.20, 95% CI: 0.05–0.92, *p* = 0.04), history of heart failure admission (cHR: 5.97, 95% CI: 1.73–20.6, *p* < 0.01), left atrium diameter (cHR: 1.08, 95% CI: 1.03–1.14, *p* < 0.01), and AF classification (cHR: 1.73, 95% CI: 1.10–2.70, *p* = 0.02). Regarding the association between hospitalized heart failure and the variables, significant associations were found in the rivaroxaban dose (cHR: 0.44, 95% CI: 0.26–0.72, *p* = 0.01), AF classification (cHR: 1.36, 95% CI: 1.07–1.73, *p* = 0.01), history of heart failure admission (cHR: 4.63, 95% CI: 2.51–8.54, *p* < 0.01), history of major bleeding (cHR: 2.31, 95% CI: 1.20–4.44, *p* = 0.01), labile INR (cHR: 2.93, 95% CI: 1.39–6.16, *p* < 0.01), vascular disease (cHR: 1.74, 95% CI: 1.05–2.87, *p* = 0.03), ticlopidine usage (cHR: 5.49, 95% CI: 1.34–22.4, *p* = 0.02), and cilostazol usage (cHR: 2.78, 95% CI: 1.01–7.65, *p* = 0.05). In the association with the clinically relevant major bleeding, the variables included rivaroxaban dose (cHR: 1.15, 95% CI: 0.56–2.38, *p* = 0.70), age (cHR: 1.10, 95% CI: 1.02–1.19, *p* = 0.02), sex (cHR: 1.07, 95% CI: 0.53–2.14, *p* = 0.56), left atrium diameter (cHR: 0.94, 95% CI: 0.90–0.98, *p* < 0.01), and history of hemorrhage stroke (cHR: 4.17, 95% CI: 1.27–13.6, *p* = 0.02). With respect to gastrointestinal bleeding, the variables in association were rivaroxaban dose (cHR: 0.57, 95% CI: 0.25–1.31, *p* = 0.19), age (cHR:1.05, 95% CI: 0.95–1.17, *p* = 0.35), sex (cHR:0.70, 95% CI: 0.30–1.62, *p* = 0.41), and usage of ticlopidine (cHR:1.66, 95% CI:1.12–2.46, *p* = 0.01). We applied the same statistical methods to assess the relationships between different study outcomes and variables among patients aged less than 85 years.

In multivariate logistic regression analyses, older patients with reduced- vs. full-dose rivaroxaban treatment were associated with a decreased risk of all-cause mortality (adjusted HR (aHR): 0.40, 95% CI: 0.21–0.74, *p* < 0.01) and hospitalized heart failure (aHR: 0.54, 95% CI: 0.29–0.99, *p* = 0.05) after adjustments were made for confounders. No significant associations were found between the rivaroxaban dose and cardiac-related mortality, clinically relevant major bleeding, and the secondary study outcomes. In the younger group, the rivaroxaban dose remained unassociated with the primary and secondary outcomes after we adjusted for confounders, except that reduced- vs. full-dose rivaroxaban was associated with a lower risk of clinically relevant bleeding complications (aHR: 0.62, 95% CI: 0.39–0.97, *p* = 0.03) and was associated with gastrointestinal bleeding at a level of borderline significance (aHR: 0.52, 95% CI: 0.26–1.03, *p* = 0.06). We show the results of the multivariate logistic regressions in Figure 3a,b.

## 4. Discussion

Treatment with reduced- vs. full-dose rivaroxaban in older patients was associated with decreased risks of all-cause mortality and hospitalized heart failure after we adjusted for confounders, but no significant associations were found with respect to safety outcomes. We showed that the efficacy of stroke prevention was unchanged in older patients with reduced- vs. full-dose rivaroxaban treatment, and the results were consistent with those of other large-scale randomized controlled trials and nationwide cohort studies [13,14]. The ELDERCARE-AF study showed that the lower dose of edoxaban was superior to the placebo in preventing stroke or systemic embolism and did not result in a significantly higher incidence of major bleeding in very elderly patients with nonvalvular atrial fibrillation [13]. Our study also showed a decreased risk of all-cause mortality in elderly individuals, and the results are supported by another nationwide study, the Taiwan National Health Insurance Research Database real-world data [14]. The study had similar results to the present study and revealed that treatment with lower-dose DOACs was significantly associated with a lower risk of mortality than treatment with full-dose DOACs in high-risk elderly individuals [14]. In contrast, another retrospective cohort study showed that Asian patients with nonvalvular atrial fibrillation aged more than 75 years had a similar hazard ratio of the net clinical benefits in lower or full rivaroxaban treatment compared with warfarin treatment [15]. Because humans currently have a longer lifespan, patients aged 75 years are maybe too young to receive reduced-dose rivaroxaban treatment. Therefore, our study used the older threshold, i.e., more than 85 years old, and the ten-year difference may contribute to the variation in study results. In addition, our study also adjusted for the effect of chronic kidney diseases on the study outcomes, but the study by Kim et al. [15] excluded patients with an estimated glomerular filtration rate less than 50 mL/min/1.73 m^2^. Therefore, our study results can be applied to clinical practice in most clinical scenarios, including normal kidney function to stage IV chronic kidney disease.

Our study showed that treatment with reduced-dose rivaroxaban was associated with a decreased risk of hospitalization for heart failure in older patients. Hospitalization for heart failure is associated with catheter ablation, antiarrhythmic agent usage, or atrial fibrillation relapse [16]. The dosage of rivaroxaban alone cannot explain the above result, so further investigation is needed.

Our study showed that treatment with reduced- vs. full-dose rivaroxaban was associated with a decreased risk of all-cause mortality and hospitalized heart failure, but the result seems partially inconsistent with previous studies, like the COMMANDER-HF study [17]. The COMMANDER-HF showed that the usage of 2.5 mg of rivaroxaban twice daily did not demonstrate a substantial decrease in the incidence of death, myocardial infarction, or stroke among patients with worsening chronic heart failure, reduced left ventricular ejection fraction, coronary artery disease, and no atrial fibrillation [17]. The difference between our study and the COMMANDER-HF study mainly exists in the patient populations; the patients in the present study had nonvalvular atrial fibrillation, but the COMMANDER-HF study enrolled patients with heart failure and reduced ejection fraction from causes other than atrial fibrillation. In addition, our patients received a higher dose of rivaroxaban (10 mg or 20 mg per day) than the patients in the COMMAND-HF study, who received a very low dose of rivaroxaban (5 mg per day). The dosage of rivaroxaban may be the other reason for the inconsistent results in our study. As mentioned above, we postulated that rivaroxaban reduces thrombus generation in patients with nonvalvular atrial fibrillation, and patients in this population may benefit from rivaroxaban treatment, whereas patients without atrial fibrillation have little thrombotic burden and cannot benefit from treatment with rivaroxaban or other DOACs. In the guidelines of Europe and the United States, rivaroxaban is available in 20/15 mg doses, but the J-ROCKET AF study has confirmed the suitability of 15/10 mg doses for use in Asian populations [10]. Previously, data from the Taiwan National Health Insurance Database indicated a lower overall mortality rate associated with the reduced doses [14], and our retrospective data have replicated this finding in real-world practice in Taiwan. Therefore, when administered following standard protocols, the reduced dose of rivaroxaban does not increase the stroke rate and may even reduce mortality.

In the younger patients, treatment with reduced- vs. full-dose rivaroxaban was not associated with any of the study outcomes, except for the decreased risk of gastrointestinal bleeding. Although systematic embolization or stroke events were not significantly different in the younger patients with reduced- vs. full-dose rivaroxaban treatment in the present study, we do not suggest the routine use of reduced-dose rivaroxaban in patients aged less than 85 years with nonvalvular atrial fibrillation. The subgroup analysis of the ROCKET-AF study showed the safety of reduced-dose rivaroxaban (15/10 mg per day) in Asian patients but, with respect to efficacy, revealed only an association of borderline significance [10]. A selection bias might exist in that the patients receiving reduced-dose rivaroxaban in the present study had a lower risk of stroke and a higher risk of bleeding complications.

Our study was a cohort study, and the major study limitation was selection bias. Physicians might prescribe reduced-dose rivaroxaban for patients with greater risks of bleeding complications, and selection bias cannot be avoided in the present study due to its inheritance. Many factors contribute to all-cause mortality, including rheumatic and tumor diseases. However, we did not record these factors in the present study, and the J-ROCKET and ELDERCARE-AF studies did not record them either. [10,13] The other major limitation was whether reduced-dose rivaroxaban therapy can be applied to Western patients with nonvalvular atrial fibrillation because the use of reduced-dose rivaroxaban was not indicated in the ROCKET-AF study [6]. In other words, our study cannot be applied to Western patients before the study results are validated externally. In the current study, we did not collect comprehensive echocardiographic data. In the next stage of our research, we intend to broaden our investigation by exploring the echocardiographic parameters in relation to the prognoses of patients with nonvalvular atrial fibrillation. Although our study has some limitations, it still has the advantage of a large sample size.

## 5. Conclusions

Reduced-dose vs. full-dose rivaroxaban treatment was associated with a decreased risk of all-cause mortality and hospitalized heart failure in elderly patients with nonvalvular atrial fibrillation, but the benefit was not a result of the decreased risks of clinically relevant bleeding complications. In contrast, younger patients receiving reduced- vs. full-dose rivaroxaban treatment did not have significantly different mortality outcomes but had a lower risk of gastrointestinal bleeding. Future studies can investigate the effect of reduced-dose rivaroxaban therapy on prognoses in Western patients.

## Figures and Tables

**Table 1:** Baseline characteristics of the patients with nonvalvular atrial fibrillation stratified by age and rivaroxaban dose.

	Age < 85 Years, *n* = 1793					Age ≥ 85 Years, *n* = 593				
	Reduced Dose		Full Dose		*p*	Reduced Dose		Full Dose		*p*
	*n* = 831		*n* = 962			*n* = 377		*n* = 216		
Baseline characteristics										
Age	74.3	7.8	71.0	9.1	<0.001	89.4	3.8	88.3	2.9	<0.001
Male gender	393	47.3%	406	42.2%	0.32	233	61.8%	117	54.2%	0.83
Atrial fibrillation type										
Paroxysmal	393	47.3%	464	48.2%	<0.001	192	50.9%	90	41.7%	<0.001
Persistent	96	11.6%	272	28.3%		30	8.0%	49	22.7%	
Permanent	69	8.3%	219	22.8%		47	12.5%	77	35.6%	
History of hospitalized heart failure	282	33.9%	368	36.3%	0.061	162	43.0%	112	51.9%	0.04
Hypertension	664	79.9%	726	75.5%	0.079	299	79.3%	162	75.0%	0.481
Uncontrolled hypertension	51	6.1%	62	6.5%	0.846	42	11.1%	13	6.0%	0.04
Diabetes mellitus	292	35.1%	369	38.4%	0.169	99	26.3%	71	32.9%	0.09
History of major bleeding complications	31	3.7%	109	11.3%	<0.001	16	4.2%	39	18.1%	<0.001
History of INR > 3	11	1.3%	88	9.2%	<0.001	5	1.3%	27	12.5%	<0.001
History of systematic thromboembolic events	202	24.3%	312	32.4%	<0.001	125	33.2%	75	34.7%	0.719
Any stroke	188	22.6%	298	31.0%	<0.001	123	32.6%	72	33.3%	0.85
History of ischemic stroke	168	20.2%	287	29.8%	<0.001	116	30.8%	69	31.9%	0.783
History of hemorrhagic stroke	25	3.0%	20	2.1%	0.228	7	1.9%	7	3.2%	0.399
CKD stage IIIb, IV, V	175	21.1%	100	10.4%	<0.001	114	30.2%	39	18.1%	0.001
Left atrium diameter (mm)	41.0	8.1	39.9	8.2	<0.001	39.7	8.2	39.5	8.4	0.792
Medication use										
Use of antiarrhythmic agents	335	40.3%	284	29.5%	<0.001	106	28.1%	51	23.6%	0.247
Types of antiarrhythmic agents										
Dronedarone	134	16.1%	51	5.3%	<0.001	55	14.6%	10	4.6%	0.001
Propafenone	60	7.2%	105	10.9%		11	2.9%	14	6.5%	
Amiodaron	121	14.6%	117	12.2%		37	9.8%	26	12.0%	
Sotalol	20	2.4%	11	1.1%		3	0.8%	1	0.5%	
Beta-blockers	298	35.9%	369	38.4%	0.351	100	26.5%	62	28.7%	0.567
Statin	294	35.4%	351	36.5%	0.657	94	24.9%	55	25.5%	0.922
Aspirin	52	6.3%	41	4.3%	0.069	5	1.3%	7	3.2%	0.133
P2Y12 inhibitor	44	5.3%	25	2.6%	0.008	8	2.1%	6	2.8%	0.589
Ticlopidine	2	0.2%	4	0.4%	0.692	2	0.5%	2	0.9%	0.625
Cilostazol	14	1.7%	11	1.1%	0.42	10	2.7%	6	2.8%	1.000
Antiplatelet or NSAID	82	9.9%	172	17.9%	<0.001	27	7.2%	46	21.3%	<0.001

## Data Availability

The study data can be provided if the correspondence judges a request is reasoable.

## Supplementary Materials

The following supporting information can be downloaded at: https://www.mdpi.com/article/10.3390/jcm12206686/s1, Table S1: Baseline characteristics in the patients with nonvalvular atrial fibrillation stratified by age. Table S2: Baseline characteristics in patients with nonvalvular atrial fibrillation stratified by rivaroxaban dose.
