# Supplementary material for: Reduced-Dose Rivaroxaban Is Associated with Lower All-Cause Mortality in Older Patients with Nonvalvular Atrial Fibrillation
Source: J Clin Med. 2023 Oct 23;12(20):6686. doi: 10.3390/jcm12206686 (PMC10607242; doi:10.3390/jcm12206686)
Supplement: Supplementary file 1 [file jcm-12-06686-s001.zip › jcm-2648518-supplementary.pdf]

Table S1. Baseline characteristics in the patients with nonvalvular atrial fibrillation stratified by age.

	Age < 85 years		Age ≧ 85 years		P
Numbers	N=1793		N=593		
Male Gender	799	44.6%	350	59%	<0.001
Atrial fibrillation type					
Paroxysmal	857	47.8%	282	47.6%	<0.001
Persistent	368	20.5%	79	13.3%	
Permanent	288	16.1%	124	20.9%	
History of hospitalized heart failure	650	36.3%	274	46.2%	<0.001
Hypertension	1390	77.5%	461	77.7%	0.902
Uncontrolled hypertension	113	6.3%	55	9.3%	0.016
Diabetes mellitus	661	36.9%	170	28.7%	<0.001
History of major bleeding complications	140	7.8%	55	9.3%	0.262
History of INR > 3	99	5.5%	32	5.4%	1.000
History of systematic thromboembolic events	514	28.7%	200	33.7%	0.023
Any stroke	486	27.1%	195	32.9%	0.007
History of ischemic stroke	455	25.4%	185	31.2%	0.006
History of hemorrhagic stroke	45	2.5%	14	2.4%	1.000
CKD stage IIIb, IV, V	275	15.3%	153	25.8%	<0.001
**Medication use**					
Use of antiarrhythmic agents	619	34.5%	157	26.5%	<0.001
Types of antiarrhythmic agents					
Dronedarone	185	10.3%	65	11.0%	<0.001
Propafenone	165	9.2%	25	4.2%	
Amiodaron	238	13.3%	63	10.6	
Sotalol	31	1.7%	4	0.7%	
Beta-blockers	667	37.2%	162	27.3%	<0.001
Statin	645	36.0%	149	25.1%	<0.001
Aspirin	93	5.2%	12	2.0%	0.001
P2Y12 inhibitor	69	3.8%	14	2.4%	0.152
Ticlopidine	6	0.3%	4	0.7%	0.277
Cilostazol	25	1.4%	16	2.7%	0.044
Antiplatelet or NSAID	254	14.2%	73	12.3%	0.271

Table S2. Baseline characteristics in patients with nonvalvular atrial fibrillation stratified by rivaroxaban dose.

	Reduced dose		Full dose		P
Numbers	N=1208		N=1178		
Male Gender	626	51.8%	523	44.4%	<0.001
Atrial fibrillation type					
Paroxysmal	585	48.4%	554	47.0%	<0.001
Persistent	126	10.4%	321	27.2%	
Permanent	116	9.6%	296	25.1%	
History of hospitalized heart failure	444	36.8%	480	40.7%	0.048
Hypertension	963	79.7%	888	75.4%	0.039
Uncontrolled hypertension	93	7.7%	75	6.4%	0.23
Diabetes mellitus	391	32.4%	440	37.4%	0.011
History of major bleeding complications	47	3.9%	148	12.6%	<0.001
History of INR > 3	16	1.3%	115	9.8%	<0.001

History of systematic thromboembolic events	327	27.1%	387	32.9%	0.002
Any stroke	311	25.7%	370	31.4%	0.002
History of ischemic stroke	284	23.5%	356	30.2%	<0.001
History of hemorrhagic stroke	32	2.6%	27	2.3%	0.6
CKD stage IIIb, IV, V	289	23.9%	139	11.9%	<0.001
Medication					
Use of antiarrhythmic agents	441	36.5%	335	28.4%	<0.001
Types of antiarrhythmic agents					
Dronedarone	189	15.6%	61	5.2%	<0.001
Propafenone	71	5.9%	119	10.1%	
Amiodaron	158	13.1%	143	12.1%	
Sotalol	23	1.9%	12	1.0%	
Beta-blockers	396	32.9%	431	36.6%	0.084
Statin	388	32.1%	406	34.5%	0.241
Aspirin	57	4.7%	48	4.1%	0.485
P2Y12 inhibitor	52	4.3%	31	2.6%	0.04
Ticlopidine	4	0.3%	8	0.5%	0.543
Cilostazol	24	2.0%	17	1.4%	0.346
Antiplatelet or NSAID	109	9.0%	218	18.5%	<0.001
